# Supplementary material for: Chromothripsis during telomere crisis is independent of NHEJ, and consistent with a replicative origin
Source: Genome Res. 2019 May;29(5):737–49. doi: 10.1101/gr.240705.118 (PMC6499312; doi:10.1101/gr.240705.118)
Supplement: Supplemental Material [file supp_gr.240705.118_Supplemental_file_1.zip › contigs/annotated_contigs/DB55/contig.2.DB55_length_362_mean_cov_4.45303867403.docx]

**DB55_length_362_mean_cov_4.45303867403**

```
ATATATTTTAATTTTTATTCCTGTTAAGCTGGGACCATTCAAAAAAGTCAACAGAAAACAGCCTAGCCTAGAAAACAGCCAGCTGTTTT
 >chrX:12771345-12771552 + E=2e-108
CCAAACTGGCTAAAACCATAAACACCTGGAAAATTATTTCTAAAAACAAAGCCAAAAAAAAATCCACAGGCCTAAGCTTTTCACTTCAG

CAAGGTTTTGGACCCACTGGTTTA|ATCC|CCAATTTACAGAAGAGAAAACTGAGGCACAGACAATTAAGAAGGTGCCCAAGTTTGTGC
                         >chrX:12719530-12719690 + E=7e-85
CCCTAATGAAGTGGAGAAACAAGTTTCAAGCCTAAGATTCCAATTCCGGCAGCCTAACTCCAGAAGCCACATTATGCTATCAATGCTGT

GTGCATTC
```
